# Review on the One-year Repeated Dose Oral Toxicity Study in Dogs for the Toxicological Evaluation of Pesticides (Agricultural Chemicals) (Decision of the Expert Committee on Pesticide, FSCJ, 21 December 2017)

**DOI:** 10.14252/foodsafetyfscj.2018008s

**Published:** 2018-12-21

**Authors:** Food Safety Commission of Japan

## Abstract

In the toxicological evaluation of pesticides (agricultural chemicals), their toxicities have been evaluated based on studies in rodents such as rats and mice as well as in non-rodents such as rabbits and dogs. Here, reflecting a research performed under the Food Safety Commission of Japan (FSCJ) grant on pesticide toxicity study^1)^, international trends and also scientific points of view, the necessity of chronic dog toxicity studies was reconsidered for the use in toxicological evaluation of pesticides.

## Conclusion

### 1. Background

In the toxicological evaluation of pesticides (agricultural chemicals), their toxicities have been evaluated based on studies in rodents such as rats and mice as well as in non-rodents such as rabbits and dogs.

The data of one-year repeated dose oral toxicity study using non-rodents (generally in dogs: refer to as a chronic toxicity in dogs hereinafter), as well as the data obtained in rodents (generally in rats), have been submitted at pesticide registration in Japan for evaluating the chronic toxicity. The data of a chronic toxicity in dogs are not mandatory for pesticide registration and others in recent abroad registration.

Here, reflecting a research performed under the Food Safety Commission of Japan (FSCJ) grant on pesticide toxicity study^1^^)^, international trends and also scientific points of view, the necessity of chronic dog toxicity studies was reconsidered for the use in toxicological evaluation of pesticides. This reconsideration shall be beneficial also for animal welfare.

Since this review is based on the scientific knowledge at present, it will be revised taking into account international trends of the toxicological evaluation procedures and new scientific knowledge.

### 2. On the Chronic Toxicity Studies in Dogs in the Toxicological Evaluation

#### (1) Fundamentals

The new approach makes it possible to perform the toxicological evaluations without the data on chronic toxicity in dogs, except for the cases described below in the section of (2).Data on chronic toxicity in dogs are, however, continually used for the evaluations in case that the corresponding data are already included in the submitted document.

During the toxicological evaluation in the pesticide committee, the committee may ask the submission of further information.

#### (2) Typical cases of necessity of chronic toxicity study in dogs

(a) Toxic profiles in dogs differ substantially from those of rodents in subacute toxicity studies.

(b) Dogs are more susceptible and a clear difference between dogs and rodents is observed in toxic doses of their identical targets.

(c) Bioaccumulation and delayed elimination are concerned in dogs on the toxicity.

(d) Distinct toxicity in dogs as shown (a) to (c) is supposedly related to the metabolism and/or toxicokinetics.

Careful considerations such as the possible relevance of the animal toxicity to humans are necessary prior to inquiring information on chronic toxicity of dogs.

In the cases obtained LOAEL but not NOAEL in subacute study and also concerned with (a) to (d), the chronic toxicity study, rather than the subacute study repeated with lower dose ranges, is recommended.
